# Buffalo Medical College

**Published:** 1852-01

**Authors:** 


*Buffalo Medical College.* Prof. Palmer.—The course on Anatomy, by Prof. Palmer, in the Medical College of Buffalo, for the present session, ended with the close of the last month. As this is the first session since the appointment of Prof. P. to the chair of anatomy, we may be permitted to say, that the expectations entertained by the friends of the institution, great as these expectations were, being based on the high reputation, as a teacher, which he has long enjoyed, have been more than fulfilled. As an able and effective teacher, Prof. P., if he have his peers, has no superior. His lectures and demonstrations are clear, accurate thorough, and are given in a manner to awaken enthusiasm in the mind of the student, secure his unremitted attention, and enforce the acquisition of anatomical knowledge.

The minute anatomy of the viscera of the abdomen and thorax forms a prominent feature of Prof. Palmer's course. This province of the study is too often overlooked, or passed over cursorily, by teachers and pupils, notwithstanding the researches of modern science have shown that an acquaintance with the elementary structure of the organs referred to is essential in order to understand physiological and pathological relations which possess great practical importance.

We do not incur the risk of running counter to the views of any who are interested in the prosperity of the Medical School at Buffalo, in congratulating the institution in having been so fortunate as to secure the services of the present incumbent of the chair of anatomy.

Prof C. A. Lee.—The course of lectures by Prof. Lee, in the Medical College of Buffalo, will commence on, or shortly after the first of the present month.

